# Normal tissue complication probability modeling for cochlea constraints to avoid causing tinnitus after head-and-neck intensity-modulated radiation therapy

**DOI:** 10.1186/s13014-015-0501-x

**Published:** 2015-09-17

**Authors:** Tsair-Fwu Lee, Shyh-An Yeh, Pei-Ju Chao, Liyun Chang, Chien-Liang Chiu, Hui-Min Ting, Hung-Yu Wang, Yu-Jie Huang

**Affiliations:** Medical Physics and Informatics Laboratory of Electronics Engineering, National Kaohsiung University of Applied Sciences, 415, Chien Kung Road, San-Min District, Kaohsiung, 80778 Taiwan ROC; Institute of Clinical Medicine, Kaohsiung Medical University, Kaohsiung, 807 Taiwan ROC; Department of Radiation Oncology, E-Da hospital, No.1, Yida Road, Jiaosu Village, Yanchao District, Kaohsiung City, 82445 Taiwan ROC; Department of Medical Imaging and Radiological Sciences, I-Shou University, Kaohsiung, 840 Taiwan ROC; Department of Radiation Oncology, Kaohsiung Chang Gung Memorial Hospital and Chang Gung University College of Medicine, Kaohsiung, 83305 Taiwan ROC

## Abstract

**Background:**

Radiation-induced tinnitus is a side effect of radiotherapy in the inner ear for cancers of the head and neck. Effective dose constraints for protecting the cochlea are under-reported. The aim of this study is to determine the cochlea dose limitation to avoid causing tinnitus after head-and-neck cancer (HNC) intensity-modulated radiation therapy (IMRT).

**Methods:**

In total 211 patients with HNC were included; the side effects of radiotherapy were investigated for 422 inner ears in the cohort. Forty-nine of the four hundred and twenty-two samples (11.6 %) developed grade 2+ tinnitus symptoms after IMRT, as diagnosed by a clinician. The Late Effects of Normal Tissues–Subjective, Objective, Management, Analytic (LENT-SOMA) criteria were used for tinnitus evaluation. The logistic and Lyman-Kutcher-Burman (LKB) normal tissue complication probability (NTCP) models were used for the analyses.

**Results:**

The NTCP-fitted parameters were *TD*_*50*_ = 46.31 Gy (95 % CI, 41.46–52.50), *γ*_*50*_ = 1.27 (95 % CI, 1.02–1.55), and *TD*_*50*_ = 46.52 Gy (95 % CI, 41.91–53.43), *m* = 0.35 (95 % CI, 0.30–0.42) for the logistic and LKB models, respectively. The suggested guideline *TD*_*20*_ for the tolerance dose to produce a 20 % complication rate within a specific period of time was *TD*_*20*_ = 33.62 Gy (95 % CI, 30.15–38.27) (logistic) and *TD*_*20*_ = 32.82 Gy (95 % CI, 29.58–37.69) (LKB).

**Conclusions:**

To maintain the incidence of grade 2+ tinnitus toxicity <20 % in IMRT, we suggest that the mean dose to the cochlea should be <32 Gy. However, models should not be extrapolated to other patient populations without further verification and should first be confirmed before clinical implementation.

## Background

Head-and-neck cancers (HNC) are some of the most serious malignancies worldwide. Radiation therapy (RT) is the mainstay of treatment, offered to almost 75 % of all HNC patients with either curative or palliative intent. Toxicities associated with RT represent important clinical outcomes that can substantially reduce the patient’s quality of life (QOL) and the ability of cancer patients to tolerate and complete the planned course of treatment [1]. During RT for HNC, the entire hearing apparatus and/or parts of the auditory system receive high doses of radiation during treatment, which can result in various radiation-induced damages to the external, middle, and inner ear [2, 3]. Morbidities associated with the inner ear include a wide range of symptoms such as tinnitus, labyrinthitis, canal paresis, vertigo/balance problems, and sensori-neural hearing loss (SNHL) [2]. Hearing loss and neurological defects have been investigated extensively. However, radiation-induced tinnitus is under-evaluated and under-reported.

The relationship between the dose of irradiation received by the cochlea and the degree of tinnitus toxicity experienced is well recognized, but poorly quantified [2, 4–6]. To our knowledge, no studies have described the normal tissue complication probability (NTCP) of the cochlea using tinnitus as the endpoint after radiation therapy. In addition, there is no Quantitative Analyses of Normal Tissue Effects in the Clinic (QUANTEC) guideline for the cochlea relating to the avoidance of tinnitus during intensity-modulated radiation therapy (IMRT). The NTCP parameters should, therefore, be determined to reveal the relationship between dose–response and radiation-induced tinnitus toxicity. Knowledge of the association between the dose distribution in the organs at risk and the probability of radiation-induced toxicity is becoming increasingly important during IMRT-planning procedures [4].

In this study, we used two common NTCP models, the logistic and the Lyman-Kutcher-Burman (LKB) models, to quantify the relationship between the incidence of tinnitus toxicity and the dose–response effects in the cochlea with the aim of identifying a specific dose relationship. The aim was to determine the best-fit parameters of these well-known and established NTCP models, and then identify the model that best describes the dose–response relationship between the cochlea and tinnitus after radiation therapy. The obtained results are likely to reveal a dose–response constraint for the cochlea that can reduce the incidence of tinnitus in radiation therapy using proper treatment planning.

## Methods

### Study population

In total 211 patients with HNC were included in this study; the side effects of radiotherapy were investigated for 422 inner ears in the cohort. All participants were treated using IMRT between June 2007 and December 2010. The characteristics of the samples are listed in Table 1. Any patients with problems related to the vestibulo-cochlear nerve were excluded from analysis because their hearing problems could be associated with pathologies of the auditory system [5, 7]. The Institutional Review Board of E-Da Hospital (EMRP-103-020) approved the study protocol, and all investigations were performed in accordance with the relevant guidelines and regulations.

**Table 1:** Characteristics of patients with head and neck cancer

	Value—x (%)
	HNC (n = 422)
Age (y)	
Mean	50
Range	26–73
Gender (*n*)	
Male	381 (90.3 %)
Female	41 (9.7 %)
Tumor site	
Larynx	48 (11.4 %)
Hypopharynx	68 (16.1 %)
Oropharynx	54 (12.8 %)
Oral cavity	102 (24.2 %)
Nasopharyngeal carcinoma	150 (35.5 %)
AJCC stage	
I	0
II	34 (8.1 %)
III	95 (22.5 %)
IV	293 (69.4 %)
Cochlea mean dose	
1–10	43 (10.2 %)
10–20	118 (28 %)
20–30	139 (32.9 %)
30–40	101 (23.9 %)
40–50	15 (3.6 %)
50–60	6 (1.4 %)
Tinnitus (grade 2+)	
Yes	49 (11.6 %)
No	373 (88.4 %)
Cochlea mean dose Gy (range)	23.72 (1.73–58.83)
Without grade 2 + Tinnitus	22.44 (1.73–50.26)
With grade 2 + Tinnitus	33.46 (8.72–58.83)
Chemotherapy	
Yes	150 (35.5 %)
No	272 (64.5 %)

*Abbreviation: AJCC* American Joint Committee on Cancer, *HNC* head and neck cancer

A basic assumption, and a potential limitation, of the present study was that the two ears of each patient could be analyzed as independent variables. Patient age and observation time were, therefore, the same for both ears, and the dose and pre-therapeutic tinnitus levels could be expected to show some correlation [8]. Based on the suggestion by Honoré *et al.* [8], the maximum-likelihood estimate of variance components was used to assess the relative importance of variation between ears and among patients in the current study.

### IMRT

Each patient’s head and shoulders were immobilized using a commercially available thermoplastic mask and/or an individually customized bite block. Computed tomography (CT) images (2.5-mm slice thickness, 512 × 512 pixels/slice) were acquired from the top of the vertex to the level of the carina (simulation CT; LightSpeed RT16, GE Medical Systems, Waukesha, WI, USA). All patients were treated using IMRT as previously described in detail [9]. The mean dose to the cochlea was kept as low as possible while still achieving the desired clinical target volume coverage. The dose distributions were calculated and dose-volume histograms (DVHs) were generated separately for each cochlea. Two IMRT techniques were used: simultaneous integrated boost (SIB), and sequential mode (SQM). Details regarding the prescribed dose and fractions for the SIB and SQM techniques can be found in previous studies [9, 10]. IMRT was delivered using the computer-controlled auto-sequencing segment or the dynamic multileaf collimator of a linear accelerator (Elekta Precise, Elekta, Crawley, UK) according to methods reported elsewhere [11]. The prescribed doses were 66.0–77.4 Gy (median, 70.0 Gy) to the macroscopic tumor (planning target volume 1 [PTV1]), 54.0–66.0 Gy (median, 61.2 Gy) to the resected tumor bed (PTV2), and 41.4–54.0 Gy (median, 50.4 Gy) to the subclinical disease area (PTV3). These were administered at 1.6–2.12 Gy per fraction using SIB, and 1.8–2.0 Gy per fraction using SQM with five fractions per week.

According to the Radiation Therapy Oncology Group 0225 and 0615 trials, the planning objectives for PTVs were a minimum dose > 95 %, and no more than 5 % of any PTV1 received ≥110 % of the prescribed dose. The mean dose constraints for the parotid gland were a mean dose ≤26 Gy or V_30Gy_ ≤50 %. For the oral cavity excluding the PTV; a mean dose ≤ 40 Gy was used. No mean dose constraints for the cochlea to avoid tinnitus have been reported previously. In most cases, the mean dose constraint to the cochlea was limited to <45 Gy. If the cochlea was adjacent to or inside the PTV1 or PTV2, the mean dose to the cochlea could be higher so as not to sacrifice the PTV coverage. The DVH values were calculated for each cochlea in all patients. All data were based on the DVHs obtained using Pinnacle^3^® (Philips, Fitchburg, WI, USA) with a bin size resolution of 0.01 Gy. The resolution of the dose calculation was 2.5 mm for all IMRT plans.

### Evaluation of tinnitus

After the completion of radiotherapy, patients were examined at 4-week intervals until their acute radiation-related complications subsided. Patients were subsequently followed-up every 2 months for the first year, and every 3 months thereafter. The evaluations performed at each follow-up visit included a medical history and physical examination. Hematology and biochemistry profiles, chest radiographs, sonography of the abdomen, a bone scan, and a CT scan of the head and neck were performed at least annually, and were checked whenever there was any clinical indication. The median follow-up period before the first detection of grade 2 tinnitus was 23 months (range: 19–37 months). The median follow-up period for the entire cohort of patients was 51 months (range: 36–77).

According to the Late Effects of Normal Tissues–Subjective, Objective, Management, Analytic (LENT-SOMA) criteria for tinnitus evaluation, the following five-point scale was used: 0, none; 1, occasional; 2, intermittent; 3, persistent; and 4, refractory [12]. Generally, grade 1 toxicities are radiographic findings of negligible clinical consequence that are rarely scored in reports of RT-induced toxicity. Grade 2 to 4 toxicities generally reflect moderate, severe, or irreversible functional damage, respectively [13].

The threshold of grade 2+ tinnitus was used as an endpoint for toxicity. The LENT-SOMA criteria for tinnitus did not define the minimum number of observations used to define the grade of toxicity. In the current study, a patient with tinnitus ≥ grade 2 on at least two observations was enrolled. Although this was a retrospective study, we assessed the occurrence of tinnitus after radiotherapy over a long period of time; the median follow-up period was 23 months. In general, a minimum follow-up period of 12 months was required before a patient could be recorded. Since there is a potential for some patients with otorrhea to develop otitis media within the first few months following radiotherapy, which would lead to temporary hearing impairment or tinnitus, such patients would be observed until the otorrhea had subsided. Any tinnitus persisting after the cessation of otorrhea would then be noted. Therefore, the hearing of all patients was evaluated carefully (including the presence of tinnitus) before beginning radiotherapy, and the history of tinnitus was reviewed to rule out symptoms that were not induced by radiation.

### Dose–response modeling

Two commonly used NTCP models, logistic and LKB [14–21], were used to quantify the relationship between the incidence of tinnitus toxicity and the dose–response effects on the cochlea.

A logistic model was used to fit the dose–response for the incidence of grade 2+ tinnitus as a function of the mean dose to the cochlea according to the following formula:

$$ NTCP=\frac{ \exp \left(4{\upgamma}_{50}\left(\frac{MD}{T{D}_{50}}-1\right)\right)}{1+ \exp \left(4{\upgamma}_{50}\left(\frac{MD}{T{D}_{50}}-1\right)\right)} $$

where *MD* is the mean dose to the cochlea; *TD*_*50*_ is the mean dose predicting a 50 % risk of complications; and *γ*_*50*_ is the normalized slope of the dose–response curve; *i.e.*, the change in NTCP per 1 % change in dose [22].

The family of Lyman-Kutcher-Burman (LKB) models is the most widely used phenomenological approach [14–21]. The LKB model is described by three parameters: *n*, *m*, and *TD*_*50*_. According to this model, NTCP is characterized by three equations:

$$ NTCP=\frac{1}{\sqrt{2\pi }}{\displaystyle {\int}_{-\infty}^t\kern0.5em }\kern0.5em  \exp \left(-{x}^2/2\right)dx $$

$$ t=\frac{D_{eff}-T{D}_{50}}{m\cdot T{D}_{50}} $$

$$ {D}_{eff}={\left({\displaystyle \sum_{i=1}^N{v}_i\cdot {D}_i^{\raisebox{1ex}{$1$}\!\left/ \!\raisebox{-1ex}{$n$}\right.}}\right)}^n $$

where *D*_*eff*_ is the dose given to the entire volume (*D*_*eff*_ is sometimes referred to as the equivalent uniform dose, EUD), *n* is a parameter that considers the volume effect, and *v*_*i*_ is the volume of the dose bin that corresponds to dose *D*_*i*_ in the differential DVH.

The parameter *m* is a unitless model parameter for describing the slope of the dose–response curve. In this study, *n* was set to 1. Therefore, *D*_*eff*_ reduces to an expression for the mean organ dose (MD). In such special cases, the LKB model can be simplified to

$$ NTCP=\frac{1}{\sqrt{2\pi }}{\displaystyle {\int}_{-\infty}^t\kern0.5em }\kern0.5em  \exp \left(-{x}^2/2\right)dx, \kern0.5em \mathrm{where}\kern0.5em t=\frac{MD-T{D}_{50}}{m\cdot T{D}_{50}} $$

The best-fit values for *TD*_*50*_, γ_50_, and *m* were identified using maximum-likelihood (ML) analysis, and the 95 % confidence intervals (CIs) were calculated using the profile-likelihood method while the parameters were fixed at the ML estimate [23]. The CIs were calculated by fixing one parameter at its best-fit value and allowing the parameter of interest to vary. The 95 % lower and upper confidence bounds were displaced downward from the ML peak by a distance determined from the chi-square distribution, *χ*^2^(0.05,1)/2 = 1.92 [24, 25]. In addition, the guideline *TD*_*20*_ was suggested as the tolerance dose that produced a 20 % complication rate within a specific period of time. All calculations were performed using the Matlab software (R2010; MathWorks, Natick, MA, USA). Although the logistic and the Lyman model yield similar NTCP parameters, they are not precisely equivalent. However, part of the aims of this study was to verify the assertion that the Lyman and logistic models are broadly similar for the same dataset.

### Performance evaluation

The model performance was measured using a variety of validation tools [26–28]. The overall performance was expressed as a scaled Brier score and checked by an Omnibus test. The scaled Brier score evaluates the differences between actual outcome and predictions, and a scaled Brier score of 1 would provide optimal agreement between predictions and actual outcomes. Model performance was also verified using the area under the receiver operating characteristic curves (AUC), and by calculating the discrimination slope, which was defined as the absolute difference between the mean predicted NTCP-values of patients with and without the evaluated endpoint [29]. The goodness of fit was further quantified in terms of calibration; *i.e.*, the agreement between the predicted and observed outcomes in the dataset, whereas the Hosmer-Lemeshow test was used to test the agreement between the expected and observed outcomes. A Hosmer-Lemeshow test value > 0.05 would show agreement between expected and observed outcomes; however, a value > 0.15 is recommended. The calibration curves were plotted to show the relationship between predicted risk and real outcome, and the curved slope of 1 showed a perfect match. The size of bin used was 10 Gy.

Akaike’s information criterion (AIC) was used to rank the accepted models. Models with smaller AIC values were considered to provide a better fit to the data than were models with larger AIC values [22]. Delta AIC (∆AIC) is a measure of each model relative to the best model. As a rule of thumb, ∆AIC = AICi – minAIC, whereas a ∆AIC >10 indicates that the model is very unlikely; values of 3–10 indicate that the model has considerably less support; values <2 suggest substantial evidence for the model and can be considered to be similar to the best model [30–32].

The negative predictive value (NPV) was also calculated, which described the rate of avoiding tinnitus when the guidelines were fulfilled based on dose-volume data. For example, NPV-*TD*_*20*_ is the fraction of all patients who had a dose below *TD*_*20*_ and who did not have toxicity. A high NPV supports the validity of a suggested guideline (*TD*_*20*_), although plans near the guideline threshold are less risky than are those distinct from the threshold (*TD*_*50*_). These analyses were performed for both *TD*_*50*_ and *TD*_*20*_ criteria. All statistical analyses were performed using the SPSS 19.0 software (SPSS, Chicago, IL, USA).

## Results

Three patients who already suffered from problems related to the vestibulo-cochlear nerve were excluded, leaving 422 samples from 211 patients to be analyzed. Forty-nine of the four hundred and twenty-two samples (11.6 %) developed grade 2+ tinnitus symptoms determined by a clinician to be caused by IMRT. The data regarding patient outcome are summarized in Table 1. The maximum-likelihood estimate of variance components revealed that there was no significant variation among the patient symptoms. This is consistent with the observations of Honoré *et al.*, and therefore the two ears of a patient could be used as independent variables of tinnitus. Average values of cochlea mean doses for patients with and without tinnitus were 33.46 Gy and 22.44 Gy, respectively. Univariate analysis was performed, which revealed a significant relationship between grade 2+ tinnitus and the mean dose to the cochlea (*p* < 0.001).

**Fig. 1:**
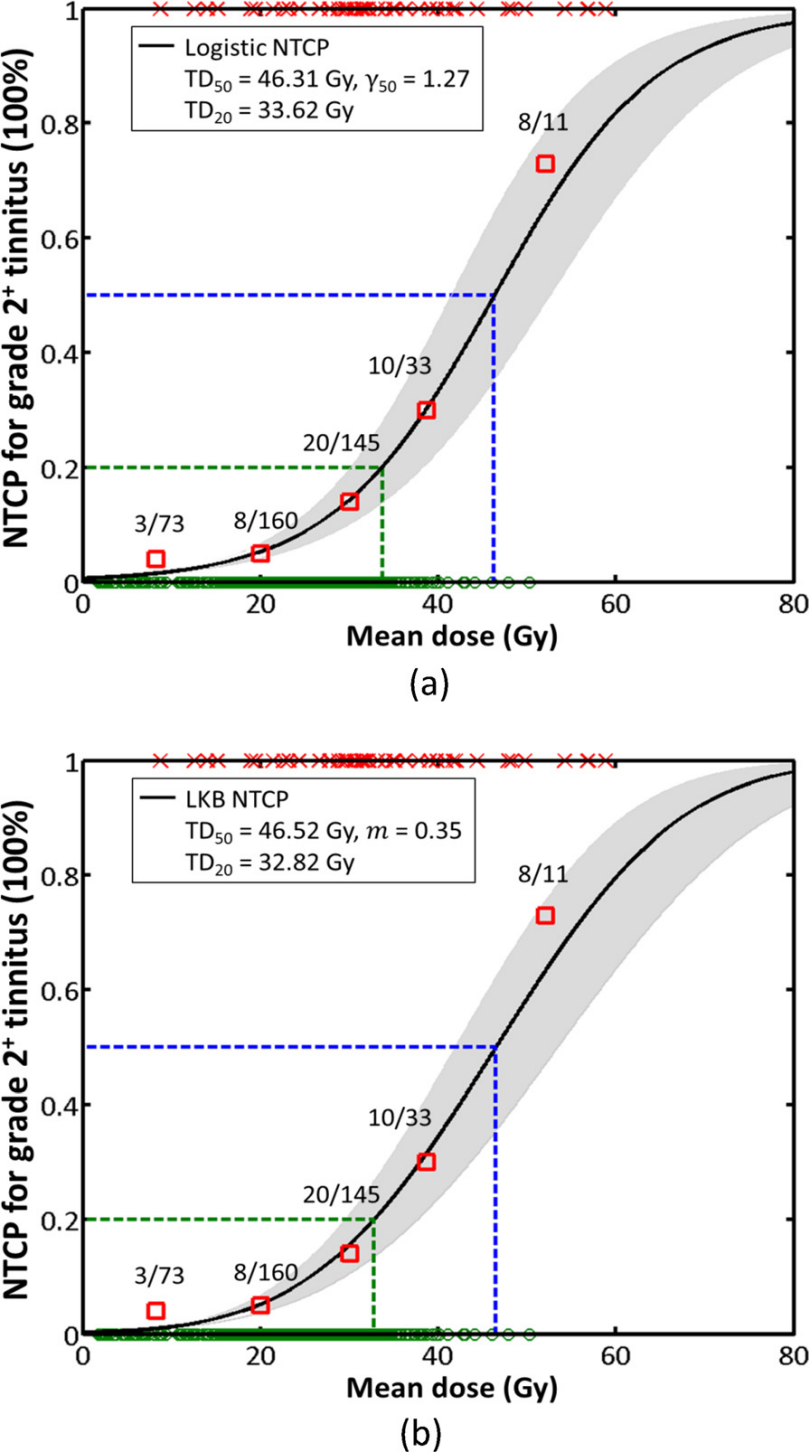
The fitted dose–response curves of the **a** logistic and **b** LKB NTCP models for the incidence of grade 2+ tinnitus. *NTCP* normal tissue complication probability, *LKB* Lyman-Kutcher-Burman

The fitted dose–response curves (logistic and LKB NTCP models) for the incidence of grade 2+ tinnitus in the HNC patient cohort are shown in Fig. 1a, b. The NTCP-fitted parameters for the logistic and LKB models were *TD*_*50*_ = 46.31 Gy (95 % CI, 41.46–52.50), *γ*_*50*_ = 1.27 (95 % CI, 1.02–1.55), and *TD*_*50*_ = 46.52 Gy (95 % CI, 41.91–53.43), *m* = 0.35 (95 % CI, 0.30–0.42), respectively (Table 2). A simple formula can be used for convenience; *i.e.*, *γ*_*50*_ ≈ 0.4/m. The odds ratio for the logistic model was 1.117 (95 % CI, 1.078–1.157). The suggested guideline *TD*_*20*_ for the tolerance dose to produce a 20 % complication rate within a specific period of time was *TD*_*20*_ = 33.62 Gy (95 % CI, 30.15–38.27) (logistic) and *TD*_*20*_ = 32.82 Gy (95 % CI, 29.58–37.69) (LKB).

**Table 2:** Normal tissue complication probability fitted parameters

NTCP model	*TD*_*50*_, Gy (95 % CI)	γ_50_ or *m* (95 % CI)	*TD*_*20*_, Gy (95 % CI)
Logistic	46.31 (41.46–52.50)	1.27 (1.02–1.55)	33.62 (30.15–38.27)
LKB	46.52 (41.91–53.43)	0.35 (0.30–0.42)	32.82 (29.58–37.69)

*Abbreviation: CI* confidence interval, *NTCP* normal tissue complication probability, *LKB* Lyman-Kutcher-Burman, *TD*_*50*_ the dose predicting a 50 % risk of complications, *TD*_*20*_ the dose predicting a 20 % risk of complications, *m* a unitless LKB model parameter for describing the slope of the dose–response curve, *γ*_*50*_ a Logistic model parameter for normalized slope of the dose–response curve

The CIs were calculated by fixing one parameter at its best-fit value and allowing the other parameter to vary

**Fig. 2:**
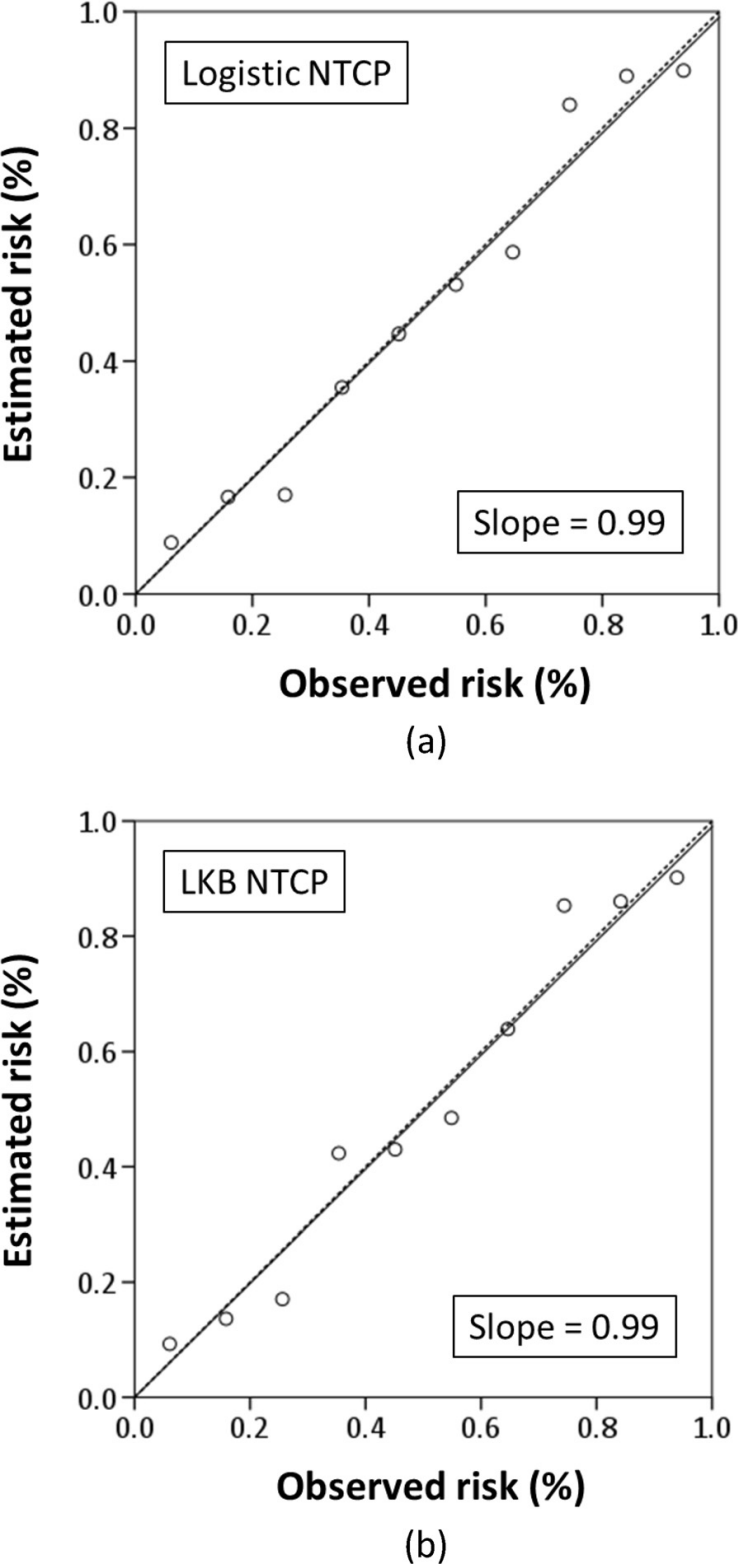
Calibration curve of the predictive models for grade 2+ tinnitus using **a** logistic and **b** LKB NTCP. The plots show the relationship between estimated risk and real outcome. *NTCP* normal tissue complication probability, *LKB* Lyman-Kutcher-Burman. Bin size = 6 Gy

The overall performance and calibration of the NTCP models for grade 2+ tinnitus toxicity according to the scaled Brier score, AUC, Hosmer-Lemeshow tests, and the calibration slope were satisfactory and within the expected ranges (Table 3). The AUC for the optimal model was 0.76 (95 % CI, 0.69–0.84) and 0.76 (95 % CI, 0.69–0.84) for the logistic and LKB NTCP models, respectively. The NPVs are provided in Table 3, and data revealed that there were very low rates of grade 2+ tinnitus. Smaller AIC values are preferable when comparing two models. However, from the point of view of ∆AIC, the logistic model is comparable with the LKB model for fitting this study dataset (∆AIC = 1.69). Finally, the calibration slope was ≥0.99 for both models, which revealed significant agreement between the predicted risk and observed outcome using both NTCP models (Fig. 2). The circles in Fig. 2 represent groups of patients with a specific mean calculated probability. The corrected NTCP is the trend line between the data points compared with the reference line, which indicates perfect calibration between predicted risk and real outcome. Overall, these data lead to the suggestion that the mean dose to the cochlea should be maintained at <32 Gy to keep the incidence of grade 2+ tinnitus toxicity at <20 % in IMRT.

**Table 3:** System performance evaluation

	Logistic NTCP	LKB NTCP
AUC	0.76 (0.69–0.84)	0.76 (0.69–0.84)
Brier (scaled)	0.16	0.16
Omnibus	P < 0.001	P < 0.001
HL test (*p-value*)	0.82	0.43
NPV-*TD*_*50*_	0.90	0.90
NPV-*TD*_*20*_	0.92	0.92
AIC	257.79	259.48

*Abbreviation: NTCP* normal tissue complication probability, *LKB* Lyman-Kutcher-Burman, *NPV* Negative predictive value, *AUC* Area under the receiver operating characteristic curve, *HL* Hosmer–Lemeshow test, *NPV* negative predictive value, *TD*_*50*_ the dose predicting a 50 % risk of complications, *TD*_*20*_ the dose predicting a 20 % risk of complications, *AIC* Akaike’s information criterion

Bin sizes used for Hosmer–Lemeshow test were 15<, 15–25, 25–35, 35–45, >45 Gy (five bins)

## Discussion

In this study, the data revealed that the occurrence of grade 2+ tinnitus after IMRT was 11.6 %. Previous studies have reported a wide range of incidences of radiation-induced tinnitus. For example, van der Putten *et al.* reported a 12 % incidence with parotid gland tumors that were treated using local or locoregional unilateral postoperative radiotherapy [4], whereas Tuan *et al.* described an incidence of 12 % in nasopharyngeal cancer using conventional radiotherapy [5]. In addition, Bakhshandeh reported an occurrence of 13.5 % in head and neck cancer treated using conventional radiotherapy [22], whereas Litre reported a much higher incidence of 64 % after the use of fractionated stereotactic radiotherapy to treat acoustic neuromas [6]. The reasons for these differences could be related to the different cancers, treatment techniques, prescription doses resulting in a difference in dose to the cochlea, or evaluation grading schemes used.

There are no current standard dose–response constraints for the cochlea because no specific NTCP values have been reported to be associated with an increased incidence of tinnitus toxicity. Quantitative Analyses of Normal Tissue Effects in the Clinic (QUANTEC), a tool resulting from recent concerted efforts among the radiotherapy community, includes reviews and summaries of normal tissue toxicity datasets and suggested dose-volume treatment planning guidelines to reduce the incidence of side effects [33]. However, there is no QUANTEC guideline for the cochlea to avoid tinnitus during IMRT. QUANTEC recommends a *TD*_*25*_ ≤ 14 Gy for SNHL for hearing at 4 kHz during the stereotactic radiosurgery for HNCs and vestibular schwannomas, and a *TD*_*30*_ ≤ 45 Gy for three-dimensional conformal radiotherapy (3D-CRT) [34]. In the current study, we suggested that the mean dose to the cochlea should be maintained at <32 Gy to maintain the incidence of grade 2+ tinnitus toxicity at <20 % (*TD*_*20*_) during IMRT.

The most serious radiation-induced complication for the inner ear is SNHL. Published studies show consistently that post-irradiation SNHL occurs in approximately 30 % of patients treated with definitive radiation using fields including the inner ear (cochlea) [12]. Although there is no agreement regarding cochlear radiation dose constraints, the minimum cochlear radiation dose reported as a risk factor for SNHL previously was 45–70 Gy [4, 8, 35, 36]. In the current study, we identified mean dose constraints of *TD*_*50*_ and *TD*_*20*_ for tinnitus; the mean dose to the cochlea must be maintained below 46 Gy if a 50 % or less probability of complication is desired. If the probability must be maintained below 20 % then the mean dose to the cochlea should be maintained below 32 Gy. These could be particularly advantageous for the treatment of HNC, when large regions require treatment and the toxicity that affects critical normal structures can have a profound impact on the patient function and quality of life [37]. *TD*_*50*_ and *TD*_*20*_ values can provide guidance for setting dose constraints and predicting the risk of grade 2+ tinnitus during treatment planning. The dose–response model described in the current study could be useful for comparing the grade 2+ tinnitus rates among different dose-fractionation schedules using IMRT for HNC.

In the current study, the dose response of the cochlea for grade 2+ tinnitus after HNC radiation therapy was used for NTCP modeling using logistic or LKB models. A goal of the current study was the retrospective enrollment of clinical data and its fitting to two different NTCP models, even though it is known that there is no difference in the fitting of logistic NTCP or LKB NTCP to the same dataset. Based on the observation that NTCP models provide a similar description of the data and the phenomenological nature of the models, the simplest model from a computational point of view could be suggested; *i.e.*, the logistic NTCP model. The logistic NTCP model also has a smaller AIC value. The results presented in the current study suggest that the clinical data could be fitted to two NTCP models to obtain similar *TD*_*50*_ values.

In the current study, we defined constraints for the mean dose to the cochlea instead of the dose-volume relationship. A dose-volume analysis is unsuitable for the cochlea because of its small volume and the limitations associated with its delineation [34]. Several studies have attempted to relate the mean or median cochlear dose to persistent hearing impairment [8, 34, 38]. All of these analyses focused on the mean dose given the small anatomical size of the cochlea. We used the maximal, mean, and minimal doses to the cochlea as the dose metrics in the NTCP models, and found that the mean dose had better correlation than did the other doses. Therefore, we selected the mean dose for use in the NTCP modeling and subsequent analyses.

Despite our observations, a large individual study sample is needed to demonstrate the independent association of these NTCP models and the risk of grade 2+ tinnitus toxicity. Moreover, treatment methods might differ among nations and institutions, and differences in radiation modalities might yield different types and levels of tinnitus toxicity. The risk of the cochlea might also be influenced by the techniques used for treatment or factors other than the dose, such as baseline patient risk factors or the co-irradiation of other organs. Therefore, these parameters should be investigated further.

## Conclusion

The dose–response limitation for the cochlea to maintain the incidence of grade 2+ tinnitus toxicity below 20 % in IMRT is that the mean dose to the cochlea should be maintained below 32 Gy. However, NTCP fits can vary based on a variety of factors, such as patient characteristics, treatment technique, follow up time, *etc.* Models should not be extrapolated to other patient populations without further verification and should be confirmed before clinical implementation.

## Abbreviations

HNC: Head and neck cancer
IMRT: Intensity-modulated radiation therapy
LKB: Lyman-Kutcher-Burman
NTCP: Normal tissue complication probability
AJCC: American Joint Committee on Cancer
CI: Confidence interval
*TD*_*50*_: the dose predicting a 50 % risk of complications
*TD*_*20*_: the dose predicting a 20 % risk of complications
*m*: a unitless LKB model parameter for describing the slope of the dose–response curve
*γ*_*50*_: a Logistic model parameter for normalized slope of the dose–response curve
RT: Radiation therapy
SNHL: Sensori-neural hearing loss
CT: Computed tomography
DVH: Dose-volume histograms
SIB: Simultaneous integrated boost
SQM: Sequential mode
PTV: Planning target volume
LENT-SOMA: the Late Effects of Normal Tissues–Subjective, Objective, Management, Analytic
NPV: Negative predictive value
AUC: Area under the receiver operating characteristic curve
HL: Hosmer–Lemeshow test
AIC: Akaike’s information criterion
